# Working towards a Co-Ordinated Approach to Invasive Mosquito Detection, Response and Control in the UK

**DOI:** 10.3390/ijerph17145166

**Published:** 2020-07-17

**Authors:** Alexander G. C. Vaux, Colin Johnston, Thom Dallimore, Liz McGinley, Clare Strode, Archie K. Murchie, Nalini Iyanger, Rachel Pudney, Yimmy Chow, Martin Brand, Ian Rea, Jolyon M. Medlock

**Affiliations:** 1 Medical Entomology and Zoonoses Ecology Group, Emergency Response Department Science and Technology, Public Health England, Porton Down, Salisbury, Wiltshire SP4 0JG, UK; Colin.Johnston@phe.gov.uk (C.J.); Liz.McGinley@phe.gov.uk (L.M.); Jolyon.Medlock@phe.gov.uk (J.M.M.); 2 Department of Biology, Edge Hill University, Ormskirk L39 4QP, UK; Dallimot@edgehill.ac.uk (T.D.); Strodecl@edgehill.ac.uk (C.S.); 3 Agri-Food and Biosciences Institute, Belfast BT9 5PX, UK; Archie.Murchie@afbini.gov.uk (A.K.M.); Ian.Rea@afbini.gov.uk (I.R.); 4 North West London Health Protection Team, Public Health England, 61 Colindale Avenue, London NW9 5EQ, UK; Nalini.Iyanger@phe.gov.uk (N.I.); Yimmy.Chow@phe.gov.uk (Y.C.); 5 Public Health England Centre South East, Health Protection—Kent Surrey & Sussex, Civic Centre, Ashford, Kent TN23 1PL, UK; Rachel.Pudney@phe.gov.uk; 6 Plant Health and Seeds Inspectorate, Animal and Plant Health Agency, Woodham Ln, Addlestone KT15 3NB, UK; Martin.Brand@apha.gov.uk

**Keywords:** *Aedes albopictus*, environmental health, mosquito control, vector-borne disease, public health

## Abstract

The United Kingdom (UK) has reported a single detection of the eggs of the invasive mosquito vector *Aedes albopictus* in each of the three years from 2016 to 2018, all in southeast England. Here, we report the detection of mosquito eggs on three occasions at two sites in London and southeast England in September 2019. Mosquito traps were deployed at 56 sites, in England, Scotland, Wales, and Northern Ireland, as part of a coordinated surveillance programme with local authorities, Edge Hill University, and government departments. Response to each detection was coordinated by Public Health England’s (PHE) local health protection teams, with technical support from PHE’s Medical Entomology group, and control conducted by the respective local authority. Control, including source reduction and larviciding, was conducted within a 300 metre radius of the positive site. The response followed a *National Contingency Plan for Invasive Mosquitoes: Detection of Incursions*. Although the response to these incidents was rapid and well co-ordinated, recommendations are made to further develop mosquito surveillance and control capability for the UK.

## 1. Introduction

In the past three decades *Aedes albopictus*, the Asian tiger mosquito, has established in Europe and expanded its range, having been reported in at least 28 countries in Europe [1,2]. Once found exclusively in forests of Southeast Asia where the principal aquatic habitats were tree holes, the species has adapted to exploit anthropogenic container habitats for breeding, particularly in urban and peri-urban landscapes, and is consequently associated with people’s homes, gardens, and yards, in water-holding containers such as tyres, buckets, litter, blocked drains, and many other water-holding habitats [1]. 

In recent years *Ae. albopictus* has established and continued its northward expansion in France, where it is now found in many major cities including Paris, Strasbourg, Lyons, as well as much of the western departments [3]. As a result of its establishment and increased abundance, coupled with the importation of travelers infected with a range of arboviruses, there have been locally acquired cases of dengue, chikungunya, and Zika in France [4,5,6,7]. In the UK, so far there have been no locally acquired cases of these mosquito-borne arboviruses due to the absence of populations of the vector. However, should *Aedes albopictus*, or any other potential vector, establish in the UK following incursions from the rest of Europe, the potential for local transmission exists. Between 2009 and 2014 the number of imported confirmed dengue cases into England, Wales, and Northern Ireland were between 160 and 550 cases [8]. For chikungunya, up to 300 confirmed cases were reported to have been imported into the UK during 2014 [9], so the potential source of virus to competent vectors exists.

In order to provide up-to-date information to direct risk surveillance for these viruses, and to prevent or delay the establishment of *Aedes albopictus* (or other invasive *Aedes* (*Stegomyia*) mosquito species) by detection and mosquito control, Public Health England (PHE) maintains a surveillance scheme aimed at identifying incursions of this mosquito [10,11] Monitoring for imported mosquitoes and subsequently mapping areas with established populations of these species is critical to minimising and managing any future arbovirus transmission in the UK. 

In 2010, invasive mosquito surveillance was set up at sites across the UK, and in 2016, eggs of *Aedes albopictus* was detected for the first time in the UK at a truck stop in Kent [12,13,14]. Eggs of this invasive mosquito were subsequently also detected in ovitraps placed in truck stops in Kent in 2017 and 2018 [11]. This paper reports on the findings of surveillance in 2019, following the same survey methodology from surveys in previous years, and with an increased number of surveillance sites [11]. The paper also details the control strategy undertaken in the event of positive detections of this species, which are now being used to guide the response and control to future incursions of these mosquitoes.

## 2. Materials and Methods

The Medical Entomology group at PHE in collaboration with Edge Hill University coordinate and support invasive mosquito surveillance activities in the UK. This involves working with local authorities, port health authorities, the Animal and Plant Health Agency (APHA) Plant Health and Seeds Inspectorate, and the Agri-Food and Biosciences Institute Northern Ireland (AFBINI) to conduct invasive mosquito surveillance and provide assistance to dealing with the response to detections.

**Figure 1:**
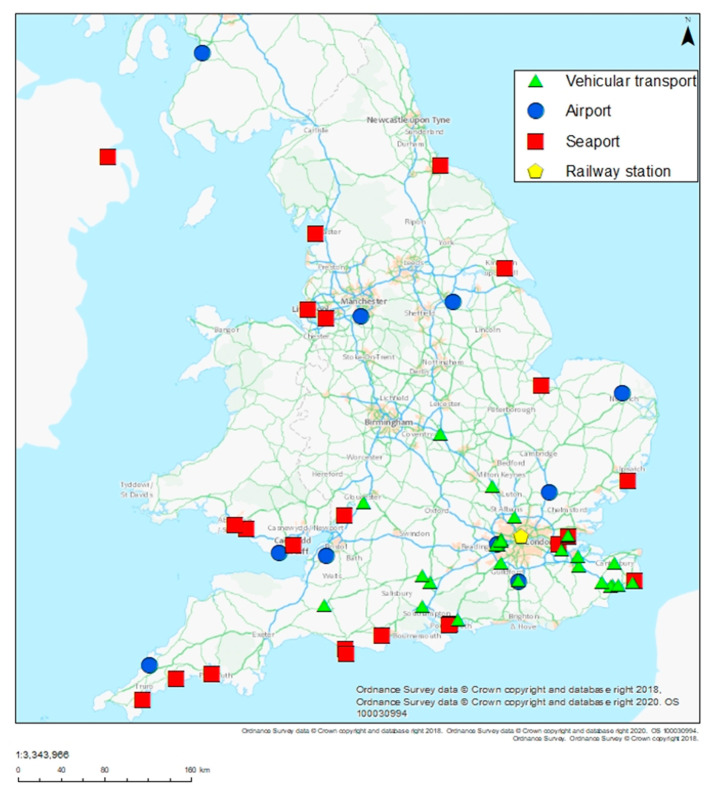
Surveillance locations shown by category: airport, seaport, railway station, vehicular transport.

Invasive mosquito surveillance was conducted at four categories of site: seaport, airports, railway stations, and vehicular transport sites (Table 1, Figure 1). In 2019, mosquito surveillance was conducted at 56 sites, from April to October at seaports and airports, and from June to October at vehicular transport sites, using Gravid *Aedes* Traps (BG-GAT^®^, Biogents, Regensburg, Germany), ovitraps, or BG-Sentinel^®^ adult traps (Biogents, Regensburg, Germany) (Table 1) [11]. Ovitraps were constructed using black plastic pots (Ramona, 11 cm in width, 9 cm in height; Luwasa^®^, Interhydro AG, Allmendingen, Switzerland), half filled with water and with a polystyrene block (5 × 5 × 5 cm) provided for oviposition. Where necessary, wire mesh was installed over ovitraps to prevent animals or birds removing the polystyrene blocks. At vehicular transport sites, traps were sited at the base of vegetation within vehicle parking areas or at the boundaries of vehicle parking areas. At goods importer sites, traps were also placed within the facility buildings where possible. Trap placement at seaports and airports varied according to local layout, but general aims were adhered to in site choice: a sheltered or undercover location; within 20 m of aircraft, baggage handling areas, or shipping containers. In general, BG-GATs were chosen at seaports and airports, as they are more resistant to disturbance by wind, people, or animals, and present less of a risk to aircraft engines than ovitraps; ovitraps were chosen at vehicular transport sites. Where possible at seaports and airports, traps were placed within the building of the border inspection posts. At St Pancras International railway station, traps were placed at the end of the platform, alongside the Eurostar trains. Where necessary, traps were housed in large dog cages to prevent tampering or the wind blowing the traps or polystyrene blocks. Traps were checked every two weeks and samples collected and sent to the laboratory at PHE Medical Entomology or at Edge Hill University. Two-week intervals between trap checks were chosen to ensure time efficiency, while ensuring sufficient checks to prevent complete larval development and adult emergence in a UK climate. Samples were identified using morphological methods (stereo microscope or scanning electron microscope) or by the sequencing of the mitochondrial cytochrome c oxidase subunit I (COI), and the nuclear internally transcribed spacer II (ITS2) genes, as described previously [11].

When invasive mosquitoes were identified, the local PHE Health Protection teams (HPT) and local authority environmental health officers (EHO) were alerted as soon as the samples were identified morphologically. On the same day, the EHOs visited the site and, in partnership with the site owners, conducted source reduction. Mosquito control focused on the site and an area of 300 m radius around the positive site, removing or treating suitable aquatic habitat with the aim of ensuring no larval development. In practice, this meant removal of litter, ensuring drains were free flowing, and treatment of drain catch basins with a silicon-based larvicide (Vazor Liquid Mosquito Film, Killgerm Ltd., Osset, UK). Vazor Liquid Mosquito Film was used according to the manufacturer’s instructions (application of 1 mL per 1 m^2^ of water surface, and retreatment after rain where necessary during a two-week period). Enhanced mosquito surveillance was initiated, which included the deployment of additional traps (10 × ovitraps; 5 × BG-GATs; 2 × BG-Sentinels) on the site and within the 300 m control area, in order to ascertain whether the positive traps were indicative of the importation of an individual *Ae. albopictus* female, or of a larger population with multiple ovi-position sites. During enhanced surveillance, traps were checked every four days by medical entomologists. Following the two-week period of enhanced surveillance, trapping was conducted fortnightly until the end of October.

## 3. Results

During 2019, *Ae. albopictus* was detected on three occasions, at two sites, all in September 2019. Polystyrene blocks positive for *Ae. albopictus* eggs were found at Ashford truck stop (1 egg at one trap) and a goods importer warehouse in Hounslow (3 eggs from two traps). Morphological identification indicated the strong likelihood that the eggs were *Ae. albopictus*, and the PHE HPT and EHO were informed; however, identification using standard DNA barcoding methods as described above was required to confirm the identity of the eggs. No larvae were found in the traps.

At the goods importer warehouse in Hounslow (vehicular transport site), two traps were positive within the warehouse, both located next to the goods inspection post utilised by the APHA Plant Health and Seeds Inspectorate. This inspection post is located approximately 15 m from the warehouse door. There were no other suitable aquatic sites within the warehouse, which included a cold storage area, and palletised goods stored on floor-to-ceiling shelving. Outside the warehouse, the immediate area is hard standing, the majority of which is for vehicle parking area and roadway. There were some areas of palletised waste cardboard storage. The only potential aquatic habitats within the 300 m control area were some drain catch basins at road kerb sides, and if they contained water, these were treated with Vazor. Enhanced surveillance was conducted using additional ovitraps, BG-GAT, and BG-Sentinel traps within the control area for two weeks from the initial finding. Mosquito control began on day one of the incident. Subsequently, no more *Ae. albopictus* eggs, larvae, or adults were found.

Ashford truck stop (vehicular transport site) supports up to 200 HGVs, is a large, rectangular area of hard standing and contains little in the way of potential larval habitat, other than rainwater drains and litter accumulating water. The site is bounded by chain-link fencing and dense vegetation (~6 m tall), and within the site boundary and the 300 metre control area, the principal aquatic habitat consisted of discarded rubbish holding rainwater. Beginning on day one of the incident, EHOs and the site owners worked together to remove rubbish on the site and within the 300 metre radius, often in densely vegetated scrubland. A small number of habitats (e.g., drain catch basin) were found to contain water and were treated with Vazor.

Thirteen days after the first detection at Ashford truck stop, 60 eggs were detected at another trap in a different location at the same site. These were identified morphologically as *Ae. albopictus*, and confirmed through DNA methods. No larvae were found in this trap. This finding took place 13 days after the initial detection at an ovitrap located 250 m away from the first positive trap. Enhanced surveillance was continued for a further two weeks, and no further evidence of *Ae. albopictus* eggs, larvae, or adults was found.

The native species *Culex pipiens* s.l., *Aedes geniculatus*, *Culiseta annulata*, and *Aedes detritus* were also recorded at the surveillance sites (Table 1).

## 4. Discussion

Following the identification of *Ae. albopictus* eggs via suspected morphological confirmation or by confirmation by molecular methods, the respective PHE HPT were informed (Ashford—PHE South East; Hounslow—PHE North West London). All identifications were confirmed through molecular methods; however, as it was necessary to enact prompt mosquito control and due to the time it took to conduct molecular tests, the HPT and EHO was alerted of the suspected finding following morphological identification. Local incidents were raised and managed through regular teleconferences, chaired by the PHE local HPT and attended by EHOs of the local authority (Ashford Borough Council and Hounslow Council, respectively), PHE Medical Entomology, and PHE Emergency Response national incident coordination team.

Incident coordination and control followed the strategy as set out in the *National Contingency Plan for Invasive Mosquitoes: Detection of Incursions* [15]. The plan sets defined levels of risk of mosquito-borne disease and invasive mosquito presence, and identifies actions accordingly. When invasive mosquitoes are detected, the response level rises from level 0 to level 1, at which point mosquito control is initiated (Table 2). At both Ashford and Hounslow sites, enhanced surveillance was conducted, and mosquito control (source reduction, and application of silicon-based larvicide) enacted, as detailed in Section 2.

The warehouse in Hounslow is typical of a goods importer warehouse in the area, receiving freight both from air cargo from nearby London Heathrow airport, as well as HGV freight from continental Europe. The area around the warehouse is of commercial and light industrial use, and used for day-time parking and vehicle loading. At this site, goods vehicles load and unload but do not park for longer periods. The finding at Ashford truck stop is the second year where *Aedes albopictus* eggs have been found at this location, the first detection being reported in 2017 [11]. The site receives a large number of trucks each day, many of which remain at the site for more than 12 h at a time. The site itself is secure, well provisioned with facilities for truck drivers, close to the motorway, and a short drive to the train and ferry, and so it is likely that truck drivers favour stopping there when arriving from France. Further findings at that location in subsequent years should be expected. The finding of additional eggs during enhanced surveillance warrants further discussion. In other studies, *Aedes albopictus* adults have been shown to disperse further than 250 m [16,17], and so the distance between the two positive traps at Ashford was not in itself sufficient to show that the findings were not related. However there was no additional evidence of invasive mosquitoes found during the enhanced surveillance period, and with the absence of adult mosquitoes or aquatic habitats with larvae present, it was concluded that the second positive trap was not related to the first and that it was not evidence of an established population, but a further separate incursion of an individual female *Ae. albopictus* mosquito.

## 5. Conclusions

In 2019, the network of surveillance sites was expanded from 46 in 2018 to 56. This was in part due to increased awareness of the issue by local authorities, and the delivery of mosquito surveillance and control training events targeting EHOs, organised by PHE Medical Entomology. It is perhaps therefore unsurprising that the surveillance programme detected three incursions at two sites in 2019, an increase from the one incursion per year in 2016–2018 [11].

It is also likely, given the increase in *Ae. albopictus* population in France and other countries in Europe, that these detections in the UK will increase. To rapidly detect and implement control measures, it is necessary to continue to target high-risk locations for importation of invasive mosquitoes, such as seaports, airports, goods warehouses, and sites supporting vehicular traffic. The organisation and delivery mechanisms following the detection of invasive mosquitoes and the subsequent control have to date been effective at coordinating a swift response, and this is in the main due to efficient, responsive PHE HPTs and engaging, experienced local authority EHOs.

However, as the situation develops over the coming years, invasive mosquitoes are likely to be found in increasingly challenging sites, such as urban centres. It is therefore necessary to make recommendations (Table 3) to develop this area of expertise and awareness among business owners, pest controllers, and EHOs in order to continue to develop a prompt, effective, control strategy at a range of sites. It is particularly important for pest controllers to have the training and tools prepared to deliver a range of mosquito control actions, including the use of both silicon-based larvicides and *Bacillus thuringiensis* subsp. *israelensis* based products, as well as the application of adulticide products to control adult mosquitoes.

## Figures and Tables

**Table 1:** Mosquito species recorded, trap type (BG-Sentinel, GAT, ovitrap), and site type (airport, seaport, vehicular transport, railway station) shown for each surveillance site. Numbers shown in brackets under trap type indicate the number of traps.

Site Type	Site Name	Trap Type	Species Received
Airport	Doncaster Airport	GAT (×10)	
Airport	Glasgow Prestwick Airport	GAT (×10)	
Airport	Manchester Airport	GAT (×10)	
Airport	Bristol Airport	GAT (×10)	
Airport	Gatwick	Ovitrap (×5) GAT (×5)	*Cx. pipiens* s.l., *Cs. annulata*
Airport	Heathrow	Ovitrap (×5) GAT (×5)	*Cx. pipiens* s.l.
Airport	Stansted	GAT (×10)	
Airport	Cardiff Airport	GAT (×10)	
Airport	Newquay Airport	GAT (×10)	*Cx. pipiens* s.l.
Airport	Norwich International Airport	GAT (×10)	*Cx. pipiens* s.l.
Seaport	Belfast Seaport	GAT (×6), Ovitrap (×6), BG-Sentinel (×1)	*Cx. pipiens* s.l., *Ae. detritus*
Seaport	Heysham Seaport and Glasson Dock	GAT (×5), Ovitrap (×5)	*Cx. pipiens* s.l.
Seaport	Hull Seaport	GAT (×10)	
Seaport	Liverpool Seaport	GAT (×10)	*Cx. pipiens* s.l.
Seaport	Manchester Seaport	GAT (×10)	
Seaport	River Tees, Middlesbrough	GAT (×10)	
Seaport	Felixstowe Seaport	GAT (×10)	
Seaport	Portsmouth Seaport	GAT (×10)	
Seaport	RN Devonport	GAT (×10)	
Seaport	RN Portsmouth	GAT (×10)	
Seaport	Tilbury Seaport	GAT (×5)	
Seaport	Weymouth Harbour	GAT (×5)	
Seaport	Portland Seaport	GAT (×5)	
Seaport	Cardiff Seaport	GAT (×5)	
Seaport	Port Talbot	GAT (×5)	
Seaport	Swansea Seaport	GAT (×5)	
Seaport	Poole Seaport	GAT (×5)	
Seaport	Falmouth Seaport	GAT (×5)	
Seaport	Fowey Seaport	GAT (×5)	
Seaport	Port Sutton Bridge	GAT (×5)	*Cx. pipiens* s.l.
Seaport	Dover Seaport	Ovitrap (×10)	*Cx. pipiens* s.l.
Seaport	London Gateway Seaport	Ovitrap (×10)	
Railway station	St Pancras International	GAT (×2)	
Vehicular transport	Canterbury Coach station	Ovitrap (×10)	*Cx. pipiens* s.l.
Vehicular transport	Goods importer A, Heathrow	Ovitrap (×4)	*Ae. albopictus* (3 × eggs)
Vehicular transport	Goods importer B, Heathrow	Ovitrap (×4)	
Vehicular transport	Fish Border Inspection Post, Heathrow	Ovitrap (×4)	
Vehicular transport	Heathrow Airport WFS Building	Ovitrap (×4)	
Vehicular transport	Gatwick Airport World Cargo Centre	Ovitrap (×4)	
Vehicular transport	Goods importer C, Heathrow	Ovitrap (×4)	
Vehicular transport	Medway	Ovitrap (×10)	
Vehicular transport	Ashford truck stop	Ovitrap (×10)	*Ae. albopictus* (1 × egg; 60 × eggs (enhanced surveillance))
Vehicular transport	Cartgate Services	Ovitrap (×10)	
Vehicular transport	Folkestone Services	Ovitrap (×10)	
Vehicular transport	Sellindge, Kent	Ovitrap (×5)	
Vehicular transport	Eurotunnel	Ovitrap (×5)	
Vehicular transport	M27 Services	Ovitrap (×10)	
Vehicular transport	A2 Services, Kent	Ovitrap (×10)	*Ae. geniculatus*
Vehicular transport	M25 Services, Elmbridge	Ovitrap (×10)	
Vehicular transport	Gloucester Services	Ovitrap (×10)	
Vehicular transport	Havant Truck stop	Ovitrap (×10)	
Vehicular transport	Maidstone Services	Ovitrap (×10)	
Vehicular transport	Watford Gap Services	Ovitrap (×10)	*Cx. pipiens* s.l.
Vehicular transport	Stobart Truck Stop	Ovitrap (×10)	
Vehicular transport	South Mimms Services	Ovitrap (×10)	*Cx. pipiens* s.l.
Vehicular transport	Toddington Services	Ovitrap (×10)	

**Table 2:** Levels of risk for invasive mosquito species and/or indigenous exotic vector-borne disease transmission (Levels 2 to 4 shown for information only). Taken from the *National Contingency Plan for Invasive Mosquitoes*.

Level 0	No invasive mosquitoes detected in England. Imported human/animal cases only.
Level 1	Invasive mosquitoes detected in an area in England. Imported human/animal cases only.
Level 2	Invasive mosquitoes established in an area in England. Imported human/animal cases only.
Level 3	Invasive mosquitoes established in an area (a) AND one or more cases of confirmed autochthonous transmission of human/animal exotic mosquito-borne infection in that area (b) OR one or more cases of confirmed autochthonous transmission of human/animal exotic mosquito-borne infection in one area.
Level 4	Invasive mosquitoes widely established in England, AND geographically spread sporadic autochthonous transmission and outbreaks of human/animal cases of an exotic mosquito-borne infection.

**Table 3:** Recommendations listed for pest controllers, environmental health, local authorities, and emergency planners.

	Recommendation
Pest controllers	Training in mosquito control applications—Identification of suitable habitats; preparedness to utilise a range of control strategies including larvicides and adulticides.
Environmental Health	Training in invasive mosquitoes (background, surveillance, and principles of control). Identification of high-risk sites in the local area. Promoting the Mosquito Recording Scheme as a mechanism for members of the general public to report nuisance biting.
Local Authorities	Incorporation of supporting Public Health England’s (PHE) mosquito surveillance scheme into routine activities. Consideration of the use of planning conditions to ensure high-risk sites are appropriately constructed and managed—e.g., the use of solid fencing to prevent mosquito dispersal; maintenance of litter-free premises. Mechanisms for enforcing access or control.
Emergency planners	Development of a local mosquito control plan.

## References

[1] Medlock J.M., Hansford K.M., Versteirt V., Cull B., Kampen H., Fontenille D., Hendrickx G., Zeller H., Van Bortel W., Schaffner F. (2015). An entomological review of invasive mosquitoes in Europe. Bull. Entomol. Res..

[2] Osório H., Zé-Zé L., Neto M., Silva S., Marques F., Silva A., Alves M. (2018). Detection of the Invasive Mosquito Species *Aedes (Stegomyia) albopictus (Diptera: Culicidae*) in Portugal. Int. J. Environ. Res. Public Health.

[3] ECDC (2019). Mosquito Maps [Internet]. European Centre for Disease Prevention and Control and European Food Safety Authority. https://ecdc.europa.eu/en/disease-vectors/surveillance-and-disease-data/mosquito-maps.

[4] ECDC (2017). Rapid Risk Assessment: Cluster of Autochthonous Chikungunya Cases in France. https://www.ecdc.europa.eu/en/publications-data/rapid-risk-assessment-cluster-autochthonous-chikungunya-cases-france.

[5] ECDC (2018). Local Transmission of Dengue Fever in France and Spain—2018. https://www.ecdc.europa.eu/en/publications-data/rapid-risk-assessment-local-transmission-dengue-fever-france-and-spain.

[6] ECDC (2019). Autochthonous Cases of Dengue in Spain and France. https://www.ecdc.europa.eu/en/publications-data/rapid-risk-assessment-autochthonous-cases-dengue-spain-and-france.

[7] ECDC (2019). Rapid Risk Assessment: Zika Virus Disease in Var Department, France. https://www.ecdc.europa.eu/en/publications-data/rapid-risk-assessment-zika-virus-disease-var-department-france.

[8] Public Health England (2015). Chikungunya in England, Wales, and Northern Ireland, 2014. https://assets.publishing.service.gov.uk/government/uploads/system/uploads/attachment_data/file/414372/Chikungunya_in_England_Wales_and_Northern_Ireland_2014.pdf.

[9] Public Health England (2015). Dengue Fever 2014. https://assets.publishing.service.gov.uk/government/uploads/system/uploads/attachment_data/file/479294/Dengue_fever_2014_FINAL_17_Nov_2015.pdf.

[10] Medlock J.M., Hansford K., Vaux A.G.C., Cull B., Gillingham E., Leach S. (2018). Assessment of the Public Health Threats Posed by Vector-Borne Disease in the United Kingdom (UK). Int. J. Environ. Res. Public Health.

[11] Vaux A.G.C., Dallimore T., Cull B., Schaffner F., Strode C., Pfluger V., Murchie A.K., Rea I., Newham Z., McGinley L. (2019). The challenge of invasive mosquito vectors in the U.K. during 2016–2018: A summary of the surveillance and control of *Aedes albopictus*. Med. Vet. Entomol..

[12] Vaux A., Murphy G., Baskerville N., Burden G., Convery N., Crossley L., Dettman L., Haden P., Jarrold L., Massey C. (2011). Monitoring for invasive and endemic mosquitoes at UK ports. Eur. Mosq. Bull..

[13] Vaux A.G.C., Medlock J.M. (2015). Current status of invasive mosquito surveillance in the UK. Parasites Vectors.

[14] Medlock J.M., Vaux A.G., Cull B., Schaffner F., Gillingham E., Pfluger V., Leach S. (2017). Detection of the invasive mosquito species *Aedes albopictus* in southern England. Lancet Infect. Dis..

[15] H.M. Government (2020). National Contingency Plan for Invasive Mosquitoes: Detection of Incursions. https://www.gov.uk/government/publications/national-contingency-plan-for-invasive-mosquitoes.

[16] Medeiros M.C.I., Boothe E.C., Roark E.B., Hamer G.L. (2017). Dispersal of male and female *Culex quinquefasciatus* and *Aedes albopictus* mosquitoes using stable isotope enrichment. PLoS Negl. Trop. Dis..

[17] Vavassori L., Saddler A., Müller P. (2019). Active dispersal of *Aedes albopictus*: A mark-release-recapture study using self-marking units. Parasites Vectors.

